# Brainstem Hemorrhage Following Eclampsia and HELLP Syndrome: A Case Report of an Unusual Neuro-Obstetric Complication

**DOI:** 10.7759/cureus.104725

**Published:** 2026-03-05

**Authors:** Marouane Jidal, Youssra Kramchi, Achraf Jeddab, Sarah Slimani, Ilyass Hmadate, Ayoub Boubekri, Rida Touab, Si mohammed Andaloussi, Khalil Mounir, Abdelhamid Jaafari

**Affiliations:** 1 Department of Surgical Intensive Care Unit, Mohammed V Military Teaching Hospital, Rabat, MAR; 2 Department of Neurology, Mohammed V Military Teaching Hospital, Rabat, MAR

**Keywords:** brainstem hemorrhage, hellp syndrome complications, posterior fossa hemorrhage, preeclampsia-eclampsia, pregnancy-related complications

## Abstract

Hypertensive disorders of pregnancy can cause severe neurological complications, including intracerebral hemorrhage. We report a peripartum case of eclampsia complicated by complete hemolysis, elevated liver enzymes, and low platelet count (HELLP) syndrome and a large posterior fossa brainstem hemorrhage with prolonged disorder of consciousness requiring prolonged intensive care and rehabilitation. Initial neuroimaging confirmed the hemorrhage with posterior fossa involvement and mass effect, and the clinical course was complicated by multiorgan dysfunction, including severe acute kidney injury requiring renal replacement therapy. This case emphasizes that persistent or unexplained coma after eclampsia should not be attributed solely to a post-ictal state or sedative effects and should prompt urgent neuroimaging to exclude catastrophic intracranial hemorrhage.

## Introduction

Hypertensive disorders of pregnancy, particularly preeclampsia and eclampsia, remain major causes of maternal morbidity and mortality worldwide and can involve multiple organs, including the brain, liver, kidneys, and the coagulation system. Neurological manifestations range from headache and visual disturbances to seizures, posterior reversible encephalopathy syndrome (PRES), and stroke [1,2]. Although pregnancy-related stroke is relatively rare (approximately 17-34 per 100,000 deliveries), its consequences for mother and fetus are often catastrophic [3,4]. In women with preeclampsia, the absolute risk of stroke remains below 1%, but preeclampsia and eclampsia are consistently identified as major risk factors for pregnancy-associated stroke and maternal death, with intracerebral hemorrhage (ICH) among the most devastating complications [1,5,6].

Cerebrovascular complications in preeclampsia and eclampsia result from widespread endothelial dysfunction, increased vascular permeability, and impaired cerebral autoregulation [7,8]. Under severe or rapidly fluctuating hypertension, the brain becomes particularly vulnerable to hyperperfusion injury, blood-brain barrier disruption, PRES, and in some cases, frank vessel rupture with ICH, especially in the posterior circulation [2,9]. Complete hemolysis, elevated liver enzymes, and low platelet count (HELLP) syndrome, a severe form of preeclampsia (hemolysis, elevated liver enzymes, and low platelet count), further increases the risk of hemorrhage via microangiopathy, thrombocytopenia, and coagulation abnormalities, and has been linked to both the occurrence and enlargement of intracranial hematomas [10,11].

Within pregnancy-associated intracerebral hemorrhage, brainstem involvement is uncommon; brainstem hemorrhage accounts for only a small minority of spontaneous ICH (≈5-10%) [12], and in the obstetric setting the available literature is largely limited to isolated case reports in women with preeclampsia with severe features (severe-range blood pressure ≥160/110 mmHg and/or end-organ dysfunction [13]) or eclampsia, often associated with HELLP syndrome and malignant hypertension. We report the case of a 37-year-old woman with preeclampsia with severe features and complete HELLP syndrome who developed a large brainstem and posterior fossa hemorrhage in the immediate peripartum period following eclampsia, adding to the limited literature on this catastrophic neuro-obstetric complication.

## Case presentation

A 37-year-old primigravida with an unmonitored pregnancy at 38 weeks and five days of gestation was admitted to a provincial hospital for preeclampsia with severe features. On admission, she was afebrile, conscious, and had no focal sensorimotor deficit. Her blood pressure was 200/130 mmHg, heart rate was 100 beats/min, and oxygen saturation was 98% on room air, with normal lung auscultation. Clinical examination also revealed bilateral lower-limb edema up to the mid-calf, headache, and blurred vision, without epigastric pain. Urinary catheterization yielded 100 mL of dark urine, and dipstick urinalysis showed 3+ proteinuria.

Initial management included two intravenous boluses of nicardipine (0.5 mg each), followed by continuous infusion at 4 mg/hr, with a target systolic blood pressure between 140 and 160 mmHg. Twenty minutes after admission, the patient developed a generalized tonic-clonic seizure consistent with eclampsia. The seizure resolved after four minutes, and intravenous midazolam (2 mg) was administered to terminate the convulsive episode. However, the patient failed to regain consciousness despite a loading dose of magnesium sulfate (4 g), followed 15 minutes later by an additional 2 g bolus.

As consciousness did not recover within 30 minutes after seizure cessation, the patient was intubated after placement of an arterial line for invasive blood pressure monitoring. A modified rapid-sequence induction was performed using fentanyl 250 µg, etomidate 30 mg, and rocuronium 80 mg. Nicardipine infusion was maintained with the same systolic blood pressure target (140-160 mmHg), and magnesium sulfate was continued as a maintenance infusion at 1 g/hr. The patient then underwent emergency cesarean section under total intravenous anesthesia with target-controlled infusion of propofol.

A live female newborn weighing 2,100 g was delivered, with Apgar scores [14] of 8, 9, and 10 at 1, 5, and 10 minutes, respectively. The newborn was monitored for 24 hours because of maternal opioid exposure before cord clamping and was subsequently discharged to the family. At follow-up, the infant remained clinically well.

Initial laboratory testing was consistent with complete HELLP syndrome, showing hemolysis documented by schistocytosis on peripheral blood smear (3%), marked thrombocytopenia, hyperbilirubinemia, and markedly elevated transaminases, together with elevated lactate dehydrogenase and markedly reduced haptoglobin (Table 1). 

**Table 1:** Key laboratory findings on admission. AST: aspartate transaminase, ALT: alanine transaminase.

Parameter	Admission value	Reference range
Hemoglobin (g/dL)	8.3	12-16
Platelet count (/mm³)	83 000	150 000-450 000
Lactate dehydrogenase (IU/L)	3 500	120-250
Haptoglobin (g/L)	<0.01	0.4-2.68
Total bilirubin (mg/L)	166	3-10
AST (IU/L)	456	<35
ALT (IU/L)	250	<40
Urea (g/L)	1.07	0.15-0.38
Creatinine (mg/l)	54	5.7-11.1

**Figure 1:**
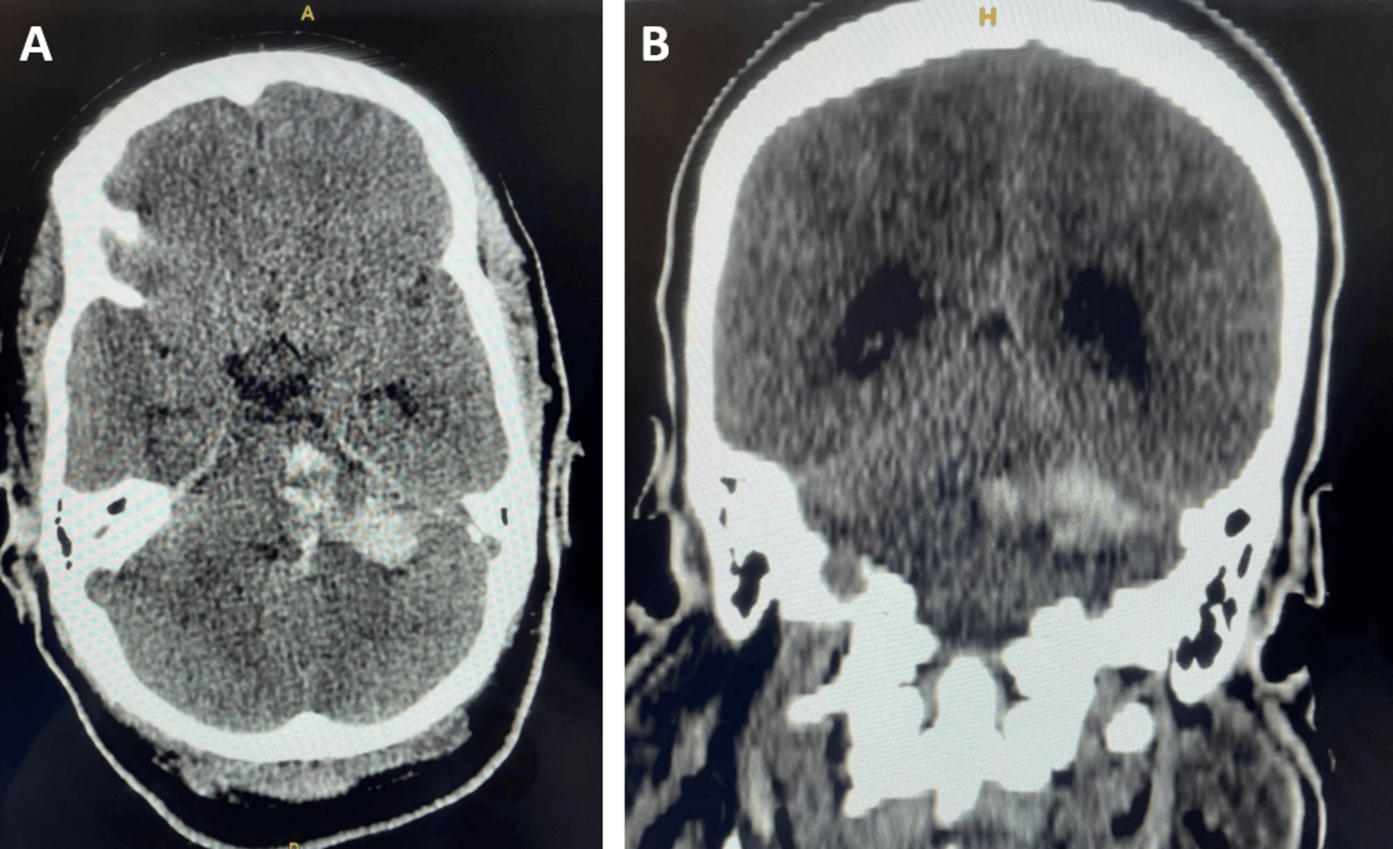
Non-contrast brain CT. Images are demonstrating an intraparenchymal brainstem hematoma extending to the left cerebellar hemisphere and left thalamus, with mass effect on the fourth ventricle, shown on (A) axial images (B) and coronal reconstruction.

An urgent non-contrast brain computed tomography (CT) scan was performed within 24 hours of the eclamptic seizure, demonstrating a pontine-predominant brainstem hematoma extending to the left thalamus and left cerebellar hemisphere, with partial compression of the fourth ventricle and associated mesencephalic herniation. The hematoma measured 32 × 46 × 42 mm (Figure 1).

**Figure 2:**
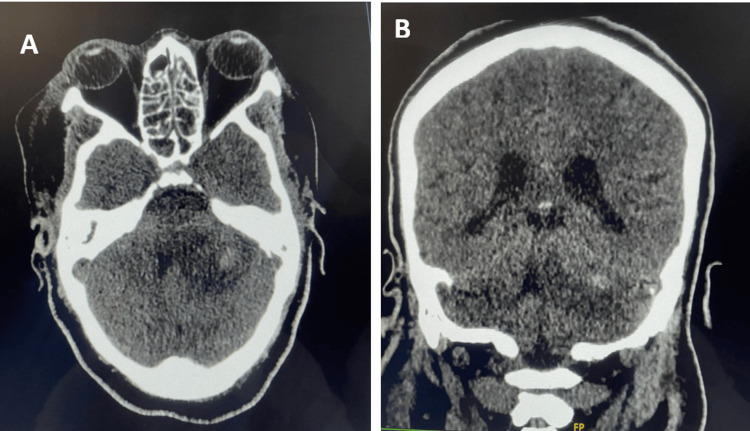
Follow-up brain CT. Images are showing liquefaction of the brainstem hematoma on (A) axial images and (B) coronal reconstruction.

The early course was complicated by acute kidney injury (KDIGO stage 3) [15] requiring several hemodialysis sessions. Despite the discontinuation of sedative agents, the patient failed to awaken after 72 hours, prompting repeat CT imaging. This showed liquefaction of the brainstem hematoma extending to the left thalamus and left cerebellar hemisphere, partially compressing the fourth ventricle and resulting in mesencephalic herniation, associated with intraventricular hemorrhage in the left lateral ventricle without hydrocephalus (Figure 2).

**Figure 3:**
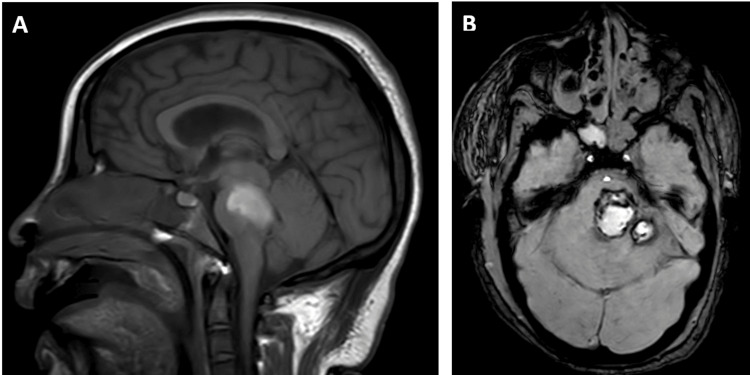
Saggital and axial MRI images. (A) Sagittal T1-weighted MRI showing an extensive hyperintense signal along the midbrain and pons (B) Axial T2*/SWI sequence demonstrating hyperintensity involving the pons, cerebral peduncle, and left cerebellar hemisphere, surrounded by a thin rim of signal void.

Given the expected prolonged ICU stay, the patient was transferred 13 days after admission to a tertiary care facility. A tracheostomy was performed two days after transfer. Hepatic and hematological parameters normalized within four days, and renal function recovered after 10 days. Despite systemic improvement, she remained with a disorder of consciousness and had a FOUR score of 8 [16]; brainstem reflexes were preserved. Brain magnetic resonance imaging (MRI) performed on admission at our center, 16 days after the eclamptic seizure, showed a late subacute hemorrhagic lesion involving the posterior fossa/brainstem with slight hemorrhagic effusion in the occipital horn of the left lateral ventricle (Figure 3).

**Figure 4:**
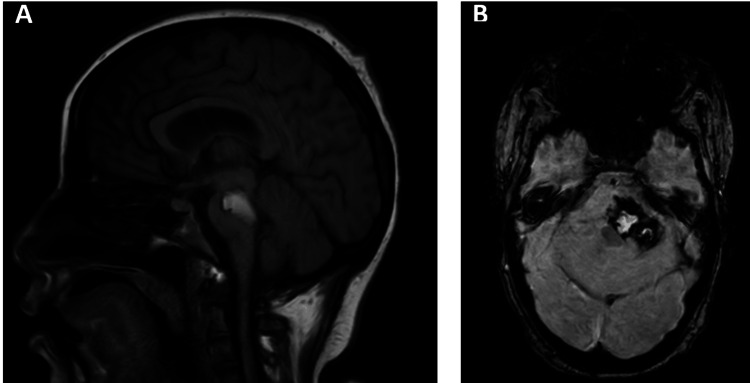
Saggital and axial images. (A) Sagittal T1-weighted MRI demonstrating a reduction in the extent of the hyperintense signal along the midbrain and pons (B) Axial T2*/SWI sequence showing a decrease in the size of the hyperintensity involving the pons, cerebral peduncle, and left cerebellar hemisphere.

Neurological status gradually improved, allowing ventilator weaning and decannulation 35 days after admission. On day 40, the Coma Recovery Scale-Revised (CRS-R) score [17] was 13/23 (A4, V4, M0, O-V2, C1, Ar2), consistent with minimally conscious state plus (MCS+). Electroencephalography showed generalized background slowing in the theta range, reactive activity, no epileptiform discharges, and intermittent light stimulation without an induced response. Follow-up MRI on day 38 showed a reduction in hematoma size with regression of perilesional edema (Figure 4).

Motor function was scored 0 on the CRS-R due to flaccid tetraplegia. Electroneuromyography performed on day 37 of admission documented absent responses in both sensory and motor nerves in all four limbs, consistent with severe ICU-acquired weakness. The patient was transferred to a specialized rehabilitation unit on day 50 after admission, with persistent neurological impairment at transfer and at the three-month follow-up.

## Discussion

Neurological complications are among the leading causes of maternal morbidity and mortality in preeclampsia and eclampsia, ranging from headaches and visual disturbances to posterior reversible encephalopathy syndrome (PRES), seizures, cerebral oedema, and both ischemic and hemorrhagic stroke [1,2]. Although relatively uncommon, intracerebral hemorrhage (ICH) is recognized as one of the most devastating neurological events in this setting [1,2,5].

Population-based data estimate the incidence of pregnancy-related stroke at around 24 per 100,000 person-years, with a relatively balanced distribution between ischemic and hemorrhagic strokes and a smaller proportion of cerebral venous thrombosis [5,18]. In women with preeclampsia, the absolute risk of stroke remains below 1%, but preeclampsia and eclampsia are consistently identified as major contributors to pregnancy-associated stroke and maternal death [1,5,6].

Most intracerebral hemorrhages reported in the setting of hypertensive disorders of pregnancy involve supratentorial regions (lobar or deep ganglionic) or the cerebellum, often in the context of severe hypertensive surges or PRES complicated by hemorrhage [2,19]. In contrast, brainstem involvement is distinctly uncommon. To contextualize this rarity, our focused review identified five published case reports of brainstem/pontine hemorrhage associated with hypertensive disorders of pregnancy [20-24], which are summarized in Table 2. These reports consistently describe severe hypertensive disease, frequent association with HELLP syndrome, and a high risk of rapid neurological deterioration and poor outcomes, including early death.

**Table 2:** Published case reports of brainstem/pontine hemorrhage associated with hypertensive disorders of pregnancy (HDP).

Study (year)	Age	Timing	HDP context	Hemorrhage site	Management	Outcome
Zeidman et al. (2005)[22]	34	H15 postpartum (40 + 5w)	HELLP	Large pontine hemorrhage + Intraventricular blood + hydrocephalus	ICU care	Death after 11 hours
Çoban et al. (2012)[23]	34	Antepartum (34 w)	Eclampsia	20x10 mm bilateral pontine hematoma with breaking into the fourth ventricle	ICU care	Neurological examination nearly normal after 6 months
Ennaqui et al. (2017)[21]	21	Day 2 postpartum (38 w)	Eclampsia	Bulbo-pontine hematoma (32*21*29mm) + Intraventricular blood	ICU care	Death after 34 hours
Ali et al. (2023)[20]	35	H15 postpartum (39 w)	Complete HELLP + Eclampsia	Large pontine hemorrhage	ICU care	WLST
Moscoso et al. (2024) [24]	33	Antepartum (21 w)	Severe preeclampsia	Bulbopontine hemorrhage (15 × 13 mm)	ICU care + obstetric curettage	NR (discharge against medical advice)
Our case	37	Antepartum (38 + 5 w)	Eclampsia + Complete HELLP	Pontine-predominant brainstem hemorrhage (32×46×42 mm)	ICU care	MCS + at 3 months

Persistent coma or evolving brainstem signs after an eclamptic seizure represent a key diagnostic pitfall. In clinical practice, depressed consciousness may be prematurely attributed to a prolonged postictal state, sedative medications administered for seizure termination, or PRES-related encephalopathy. However, the cases summarized in Table 2 illustrate that catastrophic structural lesions, particularly posterior fossa/brainstem hemorrhage with ventricular compression, can present with the same initial clinical picture. Therefore, when consciousness does not improve as expected after seizure cessation or when brainstem signs are present (e.g., pupillary abnormalities, abnormal ocular movements, impaired corneal/cough reflexes, or an unexplained respiratory pattern), urgent neuroimaging should be pursued to exclude intracranial hemorrhage, hydrocephalus, or herniation. This approach is especially relevant in patients with preeclampsia with severe features and/or HELLP syndrome, in whom severe hypertension and coagulopathy may increase the risk of hemorrhagic complications.

Our patient shared several features with previously reported cases, including severe hypertension, eclampsia, and a large pontine-predominant posterior fossa hemorrhage with mass effect (Table 2). Early CT confirmed the diagnosis and guided subsequent neurocritical care management.

Radiologically, brainstem hemorrhages in this context are typically pontine or midbrain hematomas with possible extension into the cerebellum or thalamus and frequent compromise of cerebrospinal fluid pathways, predisposing to acute obstructive hydrocephalus. The dynamic nature of the lesion (secondary enlargement of the hematoma and delayed perilesional oedema) can significantly worsen the mass effect, as illustrated in our patient. Conversely, delayed MRI in our case showed partial regression of the hematoma and surrounding oedema, in line with other conservatively managed reports [20,21].

Given the deep location of the brainstem and the high density of vital structures, there are no pregnancy-specific guidelines for brainstem hemorrhage, and management is generally individualized and largely supportive. Care is typically based on standard neurocritical care principles for intracerebral hemorrhage, including strict blood pressure control, optimization of cerebral perfusion, prevention and treatment of intracranial hypertension, and aggressive management of associated organ dysfunction. In the obstetric cases reported to date, neurosurgical procedures are uncommon and are generally reserved for selected situations such as obstructive hydrocephalus requiring cerebrospinal fluid diversion, as posterior fossa decompression carries substantial risk and uncertain benefit in this setting [25].

Several pathophysiological mechanisms may account for ICH in preeclampsia and eclampsia. These disorders are characterized by widespread endothelial dysfunction, increased vascular permeability, and impaired cerebral autoregulation [1,7-9]. Experimental and clinical data indicate that, under severe or rapidly fluctuating hypertension, autoregulatory capacity may be overwhelmed, resulting in focal hyperperfusion, blood-brain barrier disruption, PRES and, in some territories, frank vessel rupture [2,7-9,19]. The posterior circulation (vertebrobasilar system) appears particularly vulnerable, which may explain the predominance of occipital, parietal, and cerebellar involvement in neuro-obstetric presentations and, more rarely, hemorrhagic brainstem lesions. The location of the hematoma in our patient is consistent with this pattern of posterior circulation vulnerability in the setting of severe hypertension (200/130 mmHg at presentation).

HELLP syndrome adds a further layer of risk. It combines microangiopathic hemolytic anemia, substantial hepatic cytolysis, and thrombocytopenia, reflecting systemic endothelial injury and activation of coagulation pathways [10]. Thrombocytopenia and hemostatic abnormalities increase the likelihood of hemorrhage and have been linked to both the occurrence and expansion of intracranial hematomas in this context [11]. In our case, a complete HELLP syndrome with marked hemolysis, major cytolysis and platelets at 83,000/mm³ created a highly permissive milieu for vascular rupture and hematoma enlargement.

Overall, this case illustrates how the concurrence of a severe hypertensive episode in advanced preeclampsia, widespread endothelial injury with loss of cerebral autoregulation, a full-blown HELLP syndrome with thrombocytopenia, and multiorgan failure (particularly renal impairment) creates an extremely high-risk environment for ICH, even in rare and catastrophic locations such as the brainstem. This constellation of factors underscores the need for meticulous neurological surveillance, strict blood pressure control, and a low threshold for urgent neuroimaging in preeclamptic or eclamptic patients with atypical neurological symptoms or unexplained coma in the postpartum period.

## Conclusions

This case illustrates an exceptionally rare but catastrophic neuro-obstetric complication: a large brainstem and posterior fossa hemorrhage occurring in the setting of preeclampsia with severe features, complete HELLP syndrome, and multiorgan failure. The clinical course highlights the complex interplay between severe hypertension, endothelial dysfunction, coagulopathy, and acute kidney injury in creating a high-risk environment for intracerebral hemorrhage, even in anatomically critical locations such as the brainstem.

Clinically, the key teaching point is that persistent coma after an eclamptic seizure should never be presumed post-ictal; urgent brain imaging is mandatory to exclude intracranial hemorrhage and other structural complications. Early recognition of atypical neurological signs, prompt neuroimaging, rigorous blood pressure control, and aggressive management of HELLP-related hematological and organ dysfunction are essential. In young patients, timely intensive care and rehabilitation may allow partial neurological recovery, although significant residual deficits may persist. Given that this report describes a single case, broader prognostic conclusions should be interpreted cautiously and outcomes may vary across patients.
